# Antibiotic Treatment Affects Intestinal Permeability and Gut Microbial Composition in Wistar Rats Dependent on Antibiotic Class

**DOI:** 10.1371/journal.pone.0144854

**Published:** 2015-12-21

**Authors:** Monica Vera-Lise Tulstrup, Ellen Gerd Christensen, Vera Carvalho, Caroline Linninge, Siv Ahrné, Ole Højberg, Tine Rask Licht, Martin Iain Bahl

**Affiliations:** 1 Division of Diet, Disease prevention and Toxicology, National Food Institute, Technical University of Denmark, Søborg, Denmark; 2 Department of Food Technology, Engineering and Nutrition, Lund University, Lund, Sweden; 3 Department of Animal Science, Aarhus University, Tjele, Denmark; Max Rubner-Institut, GERMANY

## Abstract

Antibiotics are frequently administered orally to treat bacterial infections not necessarily related to the gastrointestinal system. This has adverse effects on the commensal gut microbial community, as it disrupts the intricate balance between specific bacterial groups within this ecosystem, potentially leading to dysbiosis. We hypothesized that modulation of community composition and function induced by antibiotics affects intestinal integrity depending on the antibiotic administered. To address this a total of 60 Wistar rats (housed in pairs with 6 cages per group) were dosed by oral gavage with either amoxicillin (AMX), cefotaxime (CTX), vancomycin (VAN), metronidazole (MTZ), or water (CON) daily for 10–11 days. Bacterial composition, alpha diversity and caecum short chain fatty acid levels were significantly affected by AMX, CTX and VAN, and varied among antibiotic treatments. A general decrease in diversity and an increase in the relative abundance of Proteobacteria was observed for all three antibiotics. Additionally, the relative abundance of *Bifidobacteriaceae* was increased in the CTX group and both *Lactobacillaceae* and *Verrucomicrobiaceae* were increased in the VAN group compared to the CON group. No changes in microbiota composition or function were observed following MTZ treatment. Intestinal permeability to 4 kDa FITC-dextran decreased after CTX and VAN treatment and increased following MTZ treatment. Plasma haptoglobin levels were increased by both AMX and CTX but no changes in expression of host tight junction genes were found in any treatment group. A strong correlation between the level of caecal succinate, the relative abundance of *Clostridiaceae* 1 family in the caecum, and the level of acute phase protein haptoglobin in blood plasma was observed. In conclusion, antibiotic-induced changes in microbiota may be linked to alterations in intestinal permeability, although the specific interactions remain to be elucidated as changes in permeability did not always result from major changes in microbiota and *vice versa*.

## Introduction

The gut microbiota is considered to have great impact on host health through either direct interaction with host cells or through production of metabolites such as short chain fatty acids [1]. Any modulation of the gut microbiota may therefore potentially lead to adverse or beneficial host effects, which may be associated to changes in intestinal integrity [2]. Many bacterial infections in humans are treated with orally administered antibiotics irrespective of the actual location of the infection. Such treatment will inevitably affect the complex and finely tuned microbial ecosystem residing in the gut [3]. Numerous studies have examined the effects of different antibiotics classes on the gut microbiota [4–11], however few have focused on the primary or secondary effects on intestinal integrity [12–14]. Intestinal permeability is a frequently employed marker for intestinal health, as increased intestinal permeability may lead to inflammation caused by bacterial components, such as lipopolysaccharide (LPS), crossing the epithelial barrier. In one study metronidazole was shown to increase the inflammatory tone of the intestine, which was linked to a disruption of the microbiota [12]. In another study high-fat diet increased intestinal permeability, but subsequent antibiotic treatment was shown to reduce the intestinal permeability again [13]. Others have shown that antibiotic treatment in childhood is associated with Crohn’s disease [15], which is connected to increased intestinal permeability [16]. Intestinal permeability in the gut is mainly controlled by the interaction between tight junction proteins linking epithelial cells. Small molecules, such as ions, are considered to pass through a high conducting ‘pore’ pathway in the tight junctions, while larger molecules, including LPS, may pass through the ‘leak’ pathway as previously defined [2,17]. Intestinal permeability, by the ‘leak’ pathway, can be assessed by determining the permeability of FITC-dextran with a defined molecular size [2,13,17].

The effect of antibiotics on the bacterial composition and consequently the intestinal permeability is expected to be dependent on the specific targets species of the antibiotics and therefore different classes of antibiotics may have different effects on intestinal health. To address this we examined effects of four antibiotics, namely; amoxicillin (AMX), cefotaxime (CTX), vancomycin (VAN), and metronidazole (MTZ) on the gut microbial composition and intestinal integrity in female Wistar rats. These antibiotics represent different classes and were chosen due to their common use for treating humans infections and varying bacterial targets (Table 1). Changes in bacterial composition were determined using high-throughput sequencing of the V3-region of the 16S rRNA encoding gene, while changes in intestinal permeability were determined *in vivo* by FITC-dextran gut permeability assay, acute phase protein haptoglobin levels in blood plasma and by measuring intestinal gene expression.

**Table 1:** Characteristics of antibiotics used in the study.

Antibiotic	Abbr.	Class	Bacterial targets [3]
Amoxicillin	AMX	Penicillin	Moderate spectrum, Gram-positives
Cefotaxime	CTX	Cephalosporin (3^rd^ gen.)	Broad-spectrum, Gram-positives and -negatives
Vancomycin	VAN	Glycopeptide	Gram-positives
Metronidazole	MTZ	Nitroimidazole	Broad-spectrum, anaerobes.

## Materials and Methods

### Ethics Statement

Animal experiments were carried out at the DTU National Food Institute (Mørkhøj, Denmark) facilities. Ethical approval was given by the Danish Animal Experiments Inspectorate. The authorization number given is 2012-15-2934-00089 C2. The experiments were overseen by the National Food Institutes in-house Animal Welfare Committee for animal care and use.

### Animals and housing

8-week old specific pathogen free female Wistar Hannover rats (n = 60) were purchased from Taconic (Lille Skensved, Denmark) and housed under controlled environmental conditions (12-hours light/dark cycles, temperature 21.5 ± 0.3°C, relative humidity 51.3 ± 3.1%, 8–10 air changes per hour). Animals had access to *ad libitum* water and feed (Altromin 1324, Altromin Spezialfutter GmbH, Germany) throughout the experiment. Animal weight, as well as feed and water intake was monitored weekly during the intervention period.

### Experimental design

Upon arrival animals were caged in pairs. The following day animals were weighed and the cages were evenly allocated into five treatment groups (six cages in each group) based on weight, and acclimatized for 2 weeks before the experimental period was initiated. Co-housing of animals was done to adhere to local animal welfare recommendations and allowed different analyses to be performed on each individual, whilst noting their non-independency. During the experimental period animals received a daily dosage of 0.5 mL of antibiotic solution (Table 1) AMX; 60 mg/mL amoxicillin (Sigma-Aldrich, A8523), CTX; 8 mg/mL cefotaxime (Sigma-Aldrich, C7912), VAN; 8 mg/mL vancomycin (Sigma-Aldrich, 861987), MTZ; 8 mg/mL Metronidazole (Sigma-Aldrich, M1547) or water (CON) by oral gavage for 10 or 11 days. Faecal pellets were collected directly from the individual rats during the trial and immediately frozen at -20°C until analysis.

### Dissection of animals

The animals that had not received FITC-dextran were dissected (n = 30). The caecum was removed, weighed, the pH of content measured (Thermo Scientific, Orion 3 Star) and samples of caecal content as well as ileum and colon content were collected and stored at -20°C. A sample of caecal content was also snap-frozen in liquid nitrogen and stores at -80°C for later short chain fatty acid analysis. Approximately 0.5–1 cm tissue samples from the colon were stored in RNAlater^®^ (Life Technologies) at -80°C for gene expression analysis.

### Extraction of bacterial community DNA

Community DNA was extracted from faecal samples collected on the initial day of dosing (Day 0), and the day before the euthanization of the first animals (Day 9), as well as from ileal and caecal content using the MoBio PowerLyzer^®^ Power Soil^®^ DNA Isolation Kit (MoBio Laboratories, Carlsbad, CA) according to the manufacturer’s recommendations with minor modifications: A maximum of 200 mg samples was used for extraction and samples were heated to 65°C for 10 min after addition of the C1 solution. Bead beating was conducted at 30 cycles/s for 4 min (Retsch MM 300 mixer mill). DNA concentrations were measured with the Qubit dsDNA HS kit (Life Technologies).

### Bacterial load

Faecal samples collected from one animal in each cage (n = 30) on Day 8 (stored at -20°C) were used for plate culturing. Ten-fold dilution series of the faecal samples were prepared in peptone saline diluent and plated on Wilkins-Chalgren agar (WCA, Oxoid). Plates were incubated at 37°C for 3 days under anaerobic conditions before enumeration. Extracted community DNA from faecal samples collected from the same animals (n = 30) on Day 9 (stored at -20°C) was used for qPCR based assessment of bacteria load based on the concentration of the 16S rRNA gene. The V3-region of the 16S rRNA gene was amplified in triplicate for each sample, using universal primers HDA1 and HDA2 (Walter et al, 2000). PCR reactions contained 5.5 μL LightCycler^®^ 480 II SYBR Green I Master (Roche Applied Science), 0.2 μM of each primer and 0.2 μL template DNA in a total reaction volume of 11 μL. Reactions conditions were: Initial 95°C for 5 minutes followed by 45 cycles of 95°C for 10 seconds, 60°C for 15 seconds and 72°C for 45 seconds. This was followed by melting curve generation (95°C for 5 seconds, 68°C for 1 minutes and increasing the temperature to 98°C with a rate of 0.11°C /second with continuous fluorescence detection). The qPCR was run in 384-well format on a LightCycler^®^ 480 II (Roche Applied Science) and analysed using the LightCycler^®^ 480 software. Tenfold dilutions of a linearized (*Sph*I-digested) plasmid standard, construction by cloning the 199bp V3 PCR amplification product of *E*. *coli* (ATCC 25922) into the pCR^®^4Blunt-TOPO vector (Invitrogen), was used for quantification of 16S rRNA genes. Additionally, the quantity of 16S rRNA genes relative to the single-copy rat host gene, TNF-α, was assessed using primers TNF_F 5’-CTGAGAGCCCCCAATCTGTG -3’ and TNF_R 5’-TCCAGTGAGTTCCGAAAGCC -3’) and the same template and reaction conditions [18].

### Amplicon sequencing of the 16S rRNA encoding gene

The bacterial composition was determined by sequencing the V3-region of the 16S rRNA gene in the extracted bacterial community DNA. Amplification of the V3-region and subsequent sequencing was performed using the Ion Torrent PGM platform (Life Technologies) as previously published [19]. Briefly, the V3-region of the 16S rRNA gene was amplified using a universal forward primer (PBU 5’-A-adapter-TCAG-barcode-CCTACGGGAGGCAGCAG-3’) with a unique 10–12 bp barcode for each bacterial community (IonXpress barcode as suggested by the supplier, Life Technologies) and a universal reverse primer (PBR 5’-trP1-adapter-ATTACCGCGGCTGCTGG-3’). The PCR reactions were conducted with 4 μL HF-buffer, 0.4μL dNTP (10mM of each base), 1 μM forward primer, 1 μM reverse primer, 5 ng template DNA, and 0.2 μL Phusion High-Fidelity DNA polymerase (Thermo Scientific) in a total reaction volume of 20 μL. Reaction conditions were as follows: Initial 98°C for 30 seconds followed by 24 cycles of 98°C for 15 seconds and 72°C for 30 seconds and finally 72°C for 5 minutes before cooling to 4°C. Products were purified by electrophoresis in 1.5% agarose gels with SYBR-safe at 3.5 V/cm for 90 min, visualized with the Safe Imager^™^ 2.0 (Invitogen) and bands with expected size of approximately 260 bp were excised from the gel. DNA was extracted using MiniElute^®^ Gel extraction kit (Qiagen) following the recommendations of the manufacturer and DNA concentrations were determined with Qubit HS assay. Finally a library was constructed by mixing an equal amount of PCR products from each original community. Sequencing was performed on a 318-chip for Ion Torrent sequencing using the Ion OneTouch^™^ 200 Template Kit v2 DL. Sequence data were obtained in FASTQ format and further processed using CLC bio genomic workbench (Qiagen) in order to de-multiplex and remove sequencing primers. Further quality trimming using default settings (remove low quality nucleotides p_base-calling error_ = 0.05, trim ambiguous nucleotides = 2) and filtering only reads with a final length between 110bp– 180bp was performed before exporting reads in FASTA format. The number of good quality reads used for taxonomical assignment ranged from 17,205 to 96,897 (except for one faecal sample, from before the intervention, that was represented by only 2,555 reads). All sequence reads were taxonomically classified using the Ribosomal Database Project Multi-classifier tool [20]. A bootstrap cut-off ≥ 50%, was chosen as recommended for fragments below 250bp and previously shown to be effective [21]. Relative abundance of bacterial taxa (at phylum and family level) were determined for each community by comparing the number of reads assigned to a specific taxa to total number of reads assigned to the bacterial root. For fold-change calculations and log transformation a relative abundance of 0.0005% analogous to 1 read in 200.000 reads was applied as a detection level. Principle component analysis (PCA) was performed on log transformed count data at the family level using the LatentiX 2.12 (Latent5; http://www.latentix.com) software package as previously described [22]. Sequencing data is deposited at NCBI Sequence Read Archive with the accession number SRP065667.

### In vivo intestinal permeability assay

Intestinal permeability was determined on the day of euthanization by measuring the permeability of FITC-dextran in fasted animals by an approach similar to previously reported [13,19]. One animal in each cage was dosed with 0.5 mL 120 mg/mL FITC-dextran (4kDa, Sigma-Aldrich, FD-4) per 100 g body weight (corresponding to 600 mg/kg animal), while the other animal was dosed with a corresponding dose of phosphate buffered saline (PBS). Exactly two hours after dosing animals were euthanized (CO_2_ and decapitation), and blood was collected from the neck into 50 ml Falcon tubes containing 100 μL EDTA (0.5M, pH 8, Ambion). Blood was centrifuged (1500 G, 10 min, 4°C), and plasma collected. Plasma was centrifuged again (5 min) before mixing 1:1 with PBS. Plasma-PBS solutions were stored dark at 5°C, until analysis the same day. Fluorescence was measured in three replicate wells for each sample (75 μL) in black 96-well microtiter plates (Proxiplate-96 F, Perkin Elmer) using a Victor TM X4 Plate reader (Perkin Elmer) with excitation at 485 nm and emission at 535 nm. Concentrations were calculated from a standard curve.

### Haptoglobin measurements

Haptoglobin was measured in blood plasma obtained from the animals, that had not received FITC-dextran (n = 30), with the "PHASE" TM Haptoglobin Assay (Tridelta Development Ltd, Kildare, Ireland,) according to the manufacturer’s recommendations. The samples were measured in duplicates and concentrations calculated from a standard curve.

### RNA extraction, cDNA preparation and Gene expression analysis

Total RNA was extracted from approximately 20 mg of colon tissue using the RNeasy Plus Mini Kit (Qiagen) and cDNA prepared from 500 ng RNA in 20 μl reactions using the SuperScript VILO cDNA Synthesis Kit (Life technologies) as previously described [19]. The relative gene expression of the tight junction proteins claudin-1, ZO-1, and occludin, as well as Mucin 2 (*Muc2*), involved in mucin production, were determined with quantitative PCR using actin beta (*Actb*) and glyceraldehyde 3-phosphate dehydrogenase (*Gapdh*) as reference genes.

### Short chain fatty acid (SCFA) analysis

Caecum content samples (approx. 200–500 mg) were thawed and transferred to 10 mL centrifuge tubes containing 3–4 glass beads (diam. 3 mm). The tubes were weighed, and a volume of 3–5 mL 0.028 M NaOH was added; the exact dilution factor was calculated for each sample. The tubes were shaken and vortexed intensively for 2 min. One milliliter suspension was transferred to a 10 mL centrifuge tube together with 100 μL internal standard (2-ethylbutyric acid, Sigma-Aldrich Denmark A/S, Vallensbæk Strand, Denmark) to a final concentration of 10 mM. Hereafter the samples were treated as previously described [23]. One milliliter of the diluted sample was extracted by adding 0.5 mL of concentrated HCl and 2 mL of diethyl ether and vortex-mixing for 30 s. After centrifugation (3,000 × *g* for 10 min), 50 μL of the ether layer was transferred to a 100 μL vial and 10 μL of the derivatization reagent N-methyl–N-t-butyldimethylsilyltrifluoroacetamide (Sigma-Aldrich Denmark A/S) was added. The reaction mixture was vortex-mixed and incubated at 80°C for 20 min, followed by a further incubation at room temperature for 48 h. The standard mixture of SCFA’s was the same as previously described [24]. Quantification of SCFA, lactic acid and succinic acid, was performed on a Hewlett Packard gas chromatograph (Model 6890, Hewlett Packard, Agilent Technologies, Naerum, Denmark) equipped with a flame-ionization detector and a 30-m ZB-5 column with an internal diameter of 0.32 mm and coated with 5%-phenyl 95%- dimethylpolysiloxane with a film thickness of 0.25 μm. The samples were injected with an auto injector (Model G 1513A, Hewlett-Packard), and the chromatograms were integrated using HP GC ChemStation software (Agilent Technologies). Detector and injector temperatures were set to 250°C. The carrier gas was helium, with a pressure of 62.6 kPa. A sample volume of 2 μL was injected with a split ratio of 20. The compounds were eluted with a temperature gradient of the following shape: held at 70°C for 3 min, increased to 110°C at 10°C / min, further increased to 290°C at 20°C / min, and held for 5 min.

### Data handling and statistics

Statistical analysis was conducted in GraphPad Prism (version 5.03; GraphPad Software Inc., La Jolla, CA) unless stated otherwise. Differences between treatment groups and the control group were assessed by the unpaired t-test or the non-parametric Mann-Whitney test as appropriate. Statistical analysis of 16S rRNA gene sequencing data was done with the web server implementation of the Metastats tool for detection of differentially abundant features (http://metastats.cbcb.umd.edu/detection.html) based on 1000 permutations and a q-value significance level of 0.05 [25]. For calculation of Shannon diversity index, sequence reads were assigned to operational taxonomic units (OTUs), using UCLUST [26] at 97% similarity threshold in QIIME v1.8 [27]. The Shannon Diversity index was calculated based on the output OTU table.

## Results

### Changes in body measurements during treatment

**Fig 1:**
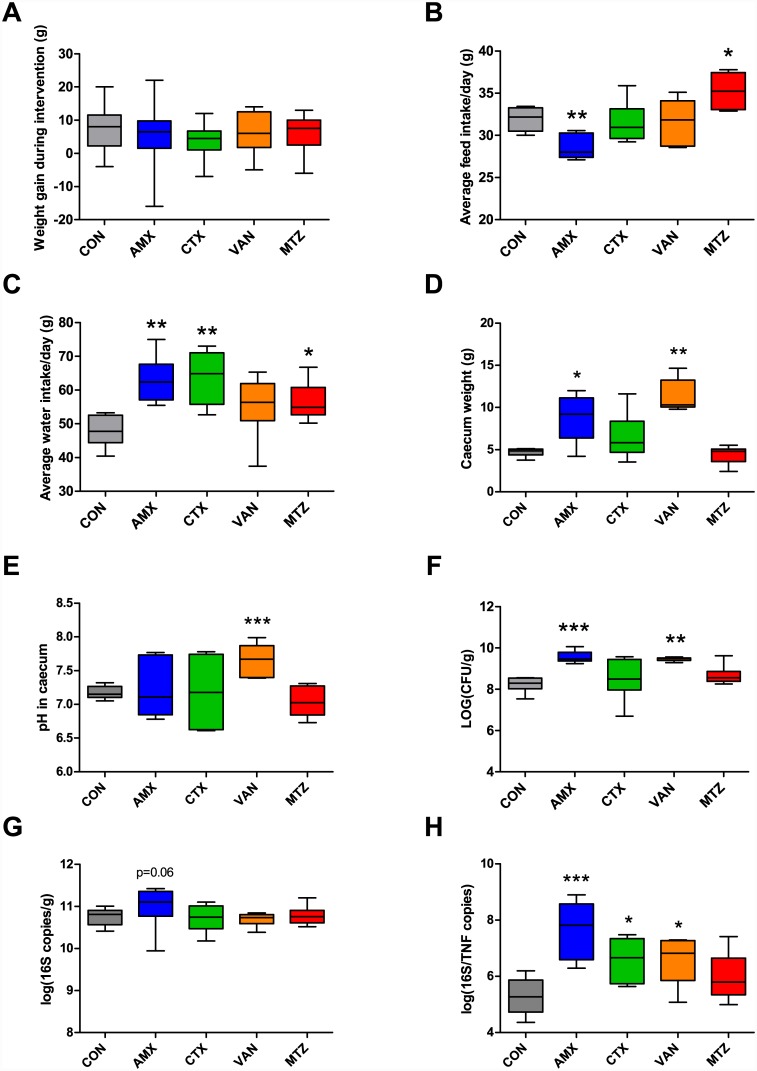
Effects of antibiotics on anthropometric measures and bacterial load. (A) Weight gain during the intervention with antibiotics (Day 0 to 9). (B) Average feed intake per day during the intervention period. (C) Average water intake per day during the intervention period. (D) Weight of caecum and (E) pH in caecum content. (F) Total anaerobic bacterial load in faecal samples determined by culturing on Wilkins-Chalgren agar. (G) Total number of 16S rRNA gene copies/g faecal sample determined by qPCR and (H) 16S rRNA gene to TNF gene ratio in faecal samples determined by qPCR. In all panels boxplots with whisker denoting the full range are shown. Significant differences between treatment groups and the control group are indicated by asterisks (*P < 0.05, **P < 0.01, ***P < 0.001). CON, control; AMX, amoxicillin; CTX, cefotaxime; VAN, vancomycin; MTZ, Metronidazole.

Animal body-weight gain did not differ between the CON group and any of the four treatment groups during the experiment (Fig 1A). Animals in the AMX group had a lower average feed intake per day during the intervention period compared to CON (P = 0.006), while animals in the MTZ group had a higher feed intake (P = 0.01) (Fig 1B). Animals in the AMX, CTX, and MTZ groups had a higher average water intake per day, than animals in the CON group (P = 0.001, P = 0.002, P = 0.02, respectively) (Fig 1C). Administration of AMX and VAN both resulted in increased caecum weight compared to CON (P = 0.04 and P = 0.002) (Fig 1D). Additionally the pH was higher in caecum content of the VAN group compared to the CON group (P = 0.0009) (Fig 1E).

### Changes in bacterial load following antibiotic treatment

The total bacterial load was determined by culture dependent as well as culture independent methods. Anaerobic culturing of faecal samples revealed significantly higher bacterial load in animals treated with AMX and VAN compared to CON, (P < 0.0001 and P = 0.005) (Fig 1F). Administration of AMX also led to higher total number of 16S copies per gram faecal content (P = 0.06) compared to CON (Fig 1G) determined by qPCR. The ratio of bacterial DNA to eukaryotic DNA, represented by copies of the 16S rRNA gene and the TNF-α host gene respectively, was significantly higher for AMX, CTX, and VAN compared to CON (Fig 1H, P = 0.0007, P = 0.01, and P = 0.01, respectively).

### Changes in bacterial composition following antibiotic treatment

**Fig 2:**
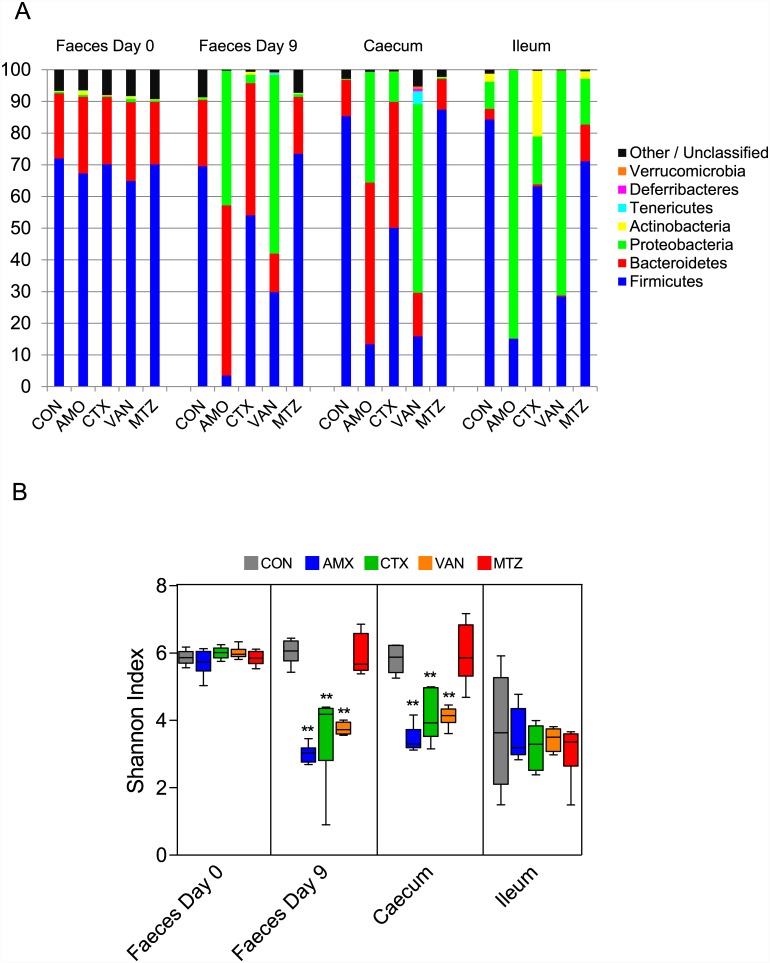
Bacterial community composition and Shannon diversity in faeces, caecum and ileum content based on 16S rRNA gene sequencing. (A) The average bacterial composition for each treatment group is shown at the phylum level for faecal samples (Day 0 and Day 9), caecal and ileal content. (B) Shannon diversity index determined in faecal samples (Day 0 and 9), caecal and ileal content for animal in CON, control (grey); AMX, amoxicillin (blue); CTX, cefotaxime (green); VAN, vancomycin (yellow) and MTZ, Metronidazole (red) groups. Boxplots with whisker denoting the full range are shown. Significant differences from CON group are indicated by asterisks (*P < 0.05, **P < 0.01).

**Fig 3:**
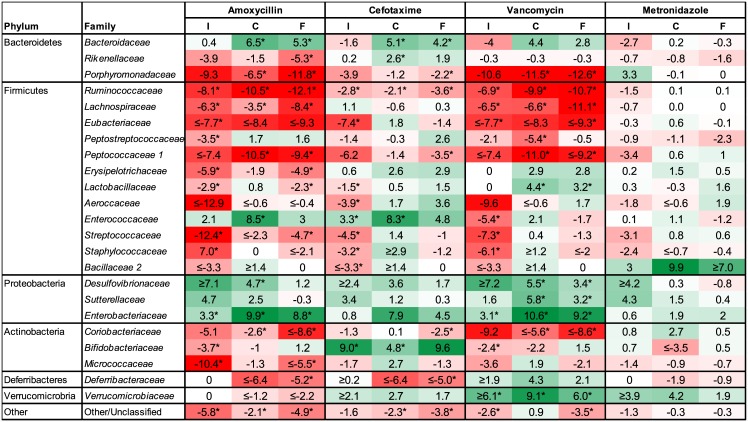
Fold change of bacterial families for treatment groups compared to the control group in ileum, caecum and faeces. Values show fold-changes (log_2_) in relative abundance (rel. abun.) of bacterial families in ileum (I), caecum (C) and faeces (F) of antibiotic treated groups (ABX) compared to the same bacterial groups in the control (CON) group (log_2_(rel. abun. ABX / rel. abun. CON)). Only bacterial families which change significantly are shown. Intensity of green and red shading indicate levels of increase or decrease respectively and asterisks indicate significant differences after correcting for multiple testing (q<0.05). When no reads were observed for a specific family, a value of 0.0005% was applied as a lower detection limit for calculations (fold-changes indicated with ≤ or ≥).

**Fig 4:**
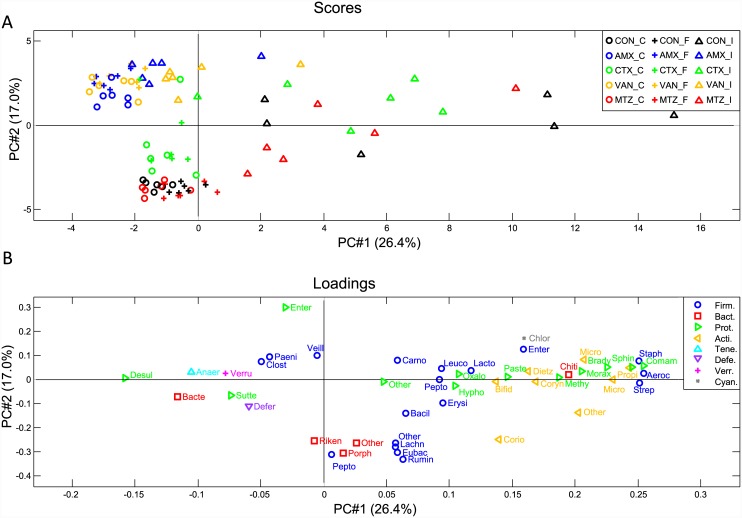
Principal component analysis (PCA) of the relative abundances of detected bacterial families in caecal, faecal and ileal samples. (A) Score plot showing samples grouped according to treatment groups CON (black), AMX (blue), CTX (green), VAN (yellow) and MTZ (red), with six animals in each group. ○ Caecal samples; + Faeces samples and Δ Ilium samples. (B) Loading plot indicating each of the bacterial families colored according to phylum. Firm, Firmicutes (blue); Bact, Bacteroidetes (red); Prot, Proteobacteria (green), Acti, Actinobacteria (yellow); Tene, Tenericutes (light blue); Defe, Deferribacteres (purple); Verr, Verrucomicrobia (lilac) and Cyan, Cyanobacteria (grey).

The bacterial composition in the AMX, CTX, and VAN groups differed significantly from the CON group in all three compartments, namely ileum content, caecum content and faecal samples at the endpoint, while MTZ did not affect the bacterial composition (Figs 2 and 3). The calculated Shannon diversity index showed a significantly lower alpha diversity for AMX, CTX and VAN in both faecal samples (Day 9) and caecum content, while no differences were found in the MTZ group (Fig 2B). Principal component analysis (PCA) showed that AMX and VAN treatment resulted in clustering of samples from all three compartments (ileum, caecum and faeces), while only faecal and caecal samples clustered together in the CTX, MTZ and CON groups (Fig 4). Each of the antibiotic treatment groups clustered differently from the others when including only caecal and faecal samples in the PCA (S1 Fig). No difference was observed between MTZ and CON, however PCA modeling based on these two groups alone did show a slight difference for caecal samples (data not shown). The loading plot (Fig 4B) indicated that Proteobacteria and specifically *Enterobacteriaceae* may be driving the shift towards a higher PC#2 score for antibiotics AMX, VAN and CTX. Generally the ileum samples showed a lot of variation within groups, however it was observed that both AMX and VAN treatment reduced beta diversity (S2 Fig). The average bacterial community compositions in faecal samples taken before treatment (faeces Day 0) were similar for all groups (Fig 2).

Overall, the bacterial composition in samples from all three compartments (ileal, caecal and faecal samples) within the same treatment group changed in the same direction as compared to CON (Fig 3). The relative abundance of families within the Firmicutes and Actinobacteria phyla were generally reduced following treatment with AMX in all three compartments, while families within the Proteobacteria were increased (Figs 2A and 3). Within the Bacteroidetes, an increase in the relative abundance of *Bacteroidaceae* and a decrease in both *Rikenellaceae* and *Porphyromonadaceae* following AMX treatment was noted. The response of the bacterial community to VAN was similar to AMX, however the *Verrucomicrobiaceae* were significantly increased (in all three compartments) only after VAN treatment. Treatment with CTX resulted in more subtle changes of the community structure than observed for AMX and VAN (Fig 3), including significant increases of relative abundances of *Bacteroidaceae*, *Rikenellaceae* and *Enterococcaceae* and reductions in relative abundances of *Porphyromonadaceae*, *Ruminococcaceae* and *Peptococcaceae 1*. Nevertheless, CTX was the only antibiotic causing a significant increase in *Bifidobacteriaceae* (Fig 3).

### Intestinal permeability

**Fig 5:**
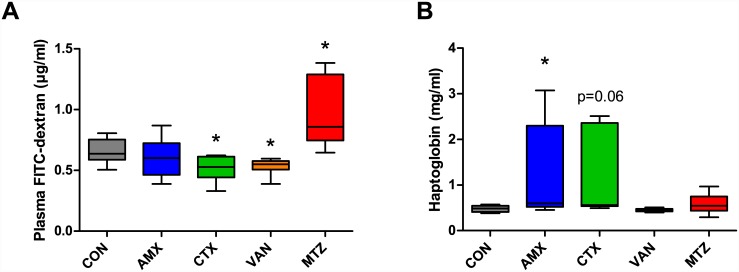
Blood plasma measurements. (A) Plasma FITC-dextran concentrations and (B) Haptoglobin concentration in blood plasma. Boxplots with whisker denoting the full ranges are shown. CON, control (grey); AMX, amoxicillin (blue); CTX, cefotaxime (green); VAN, vancomycin (yellow); MTZ, Metronidazole (red). Significant differences from CON group are indicated by asterisks (*P < 0.05).

Antibiotic treatment with CTX or VAN resulted in lower FITC-dextran plasma concentrations compared to CON (P = 0.04, and P = 0.04, respectively) following oral administration, indicating decreased intestinal permeability (Fig 5A). Oppositely, administration of MTZ increased the plasma FITC-dextran concentration (P = 0.02) compared to CON (Fig 5A). The acute phase protein haptoglobin level was increased in the AMX group (P = 0.03) and a trend of increase was seen in the CTX group (P = 0.06) compared to the control group (Fig 5B). These differences were driven by two out of six animals in the AMX as well as in the CTX group, which had levels of haptoglobin that were approximately four times higher than the mean value of the CON group.

No significant difference in the relative expression of ZO-1, occludin, Claudin-1 or *Muc2* were found between the CON group and any of the antibiotic treatment groups (S2 Fig)

### Changes in short chain fatty acids and succinate in caecum

**Fig 6:**
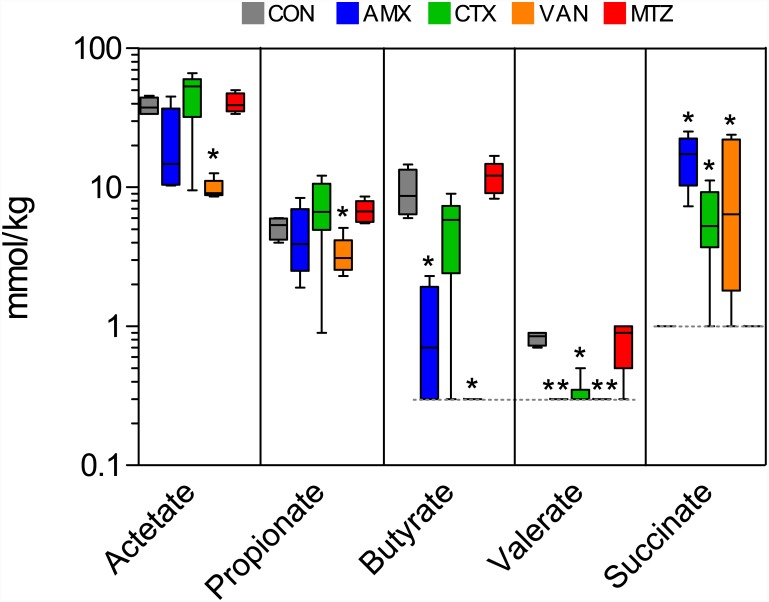
Short chain fatty acid and succinate. Concentrations of acetate, propionate, butyrate, valerate and succinate in caecum for each treatment group. Boxplots with whisker denoting the full range are shown. Control (grey), amoxicillin (blue), cefotaxime (green), vancomycin (yellow) and metronidazole (red). The dashed lines indicate limit of detection. Significant differences from CON group are indicated by asterisks (*P < 0.05, **P < 0.01).

Treatment with the different classes of antibiotics resulted in changes in caecum levels of SCFA and succinate compared to the CON group (Fig 6). Acetate and propionate levels were both lower in the VAN group (P = 0.02 and P = 0.03), butyrate was lower in the AMX and VAN groups (both P = 0.01), valerate was lower in the AMX, CTX and VAN groups (P = 0.006, P = 0.009 and P = 0.01) and succinate was higher in the AMX, CTX and VAN groups (P = 0.01, P = 0.03 and P = 0.04) compared to the CON group. No statistically significant differences in SCFA’s and succinate levels were observed for the MTZ group.

## Discussion

As expected, administration of AMX, CTX and VAN for ten days resulted in major shifts in the microbial communities and reduction of Shannon diversity in caecal and faecal samples (Figs 2, 3 and 4). The shifts in community structure were distinct for the different classes of antibiotics with close clustering of samples within treatment group (S1 Fig). No effects on the microbiota were seen following treatment with MTZ, which is probably because only low concentrations of active metronidazole reach the ileum, caecum and colon, as this antibiotic is well absorbed in the small intestine [28]. Although antibiotic treatment is generally anticipated to reduce bacterial loads, cultivation-based and molecular methods both revealed an increase in the total bacterial loads after treatment with AMX or VAN (Fig 1F–1H). This was presumably caused by expansion of intrinsically resistant bacterial groups to occupy new niches within the gut environment, and has previously been noted in other studies [9].

**Fig 7:**
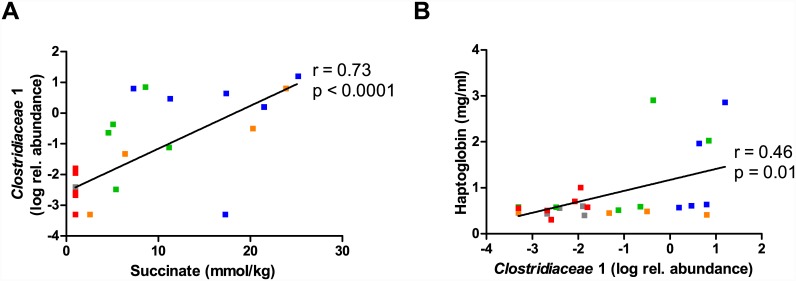
Correlation analysis. (A) Spearman correlation between succinate and the relative abundance of *Clostridiaceae* 1 family and (B) between the relative abundance of *Clostridiaceae* 1 family and plasma haptoglobin levels. Each dot represents individual animals within the CON, control (grey); AMX, amoxicillin (blue); CTX, cefotaxime (green); VAN, vancomycin (yellow); MTZ, Metronidazole (red) groups and lines show the linear regression. Spearman r and P values are shown.

Treatment with AMX, CTX and VAN resulted in changes in SCFA levels as well as increased levels of succinate in the caecum, which was undetectable in the CON group (Fig 6). Increased succinate could be attributed to the increase in relative abundance of *Bacteroidaceae* that includes species known for succinate production [29]. A streptomycin-induced increase in succinate levels has recently been shown to facilitate expansion of the human pathogen *Clostridium difficile* (*Peptostreptococcaceae* family) in the mouse gut [30]. In the present study we did not observe this effect at the family level, but on the contrary found a decrease in the relative abundance of *Peptostreptococcaceae* in the ileum and caecum of animals treated with AMX and VAN, respectively (Fig 3). We did however find a strong correlation (r = 0.73, p < 0.0001) between the caecum succinate concentration and the relative abundance of the *Clostridiaceae* 1 family (Fig 7A). This family of Clostridiales contains the genus *Clostridium sensu stricto* with notable pathogenic member species (e.g. *C*. *botulinum* and *C*. *perfringens*) and the finding could thus signify that also other potentially pathogenic bacteria may propagate due to elevated succinate levels caused by the antibiotic induced microbial imbalance. We further found a significant positive correlation between the relative abundance of the *Clostridiaceae* 1 family and the plasma acute phase protein haptoglobin level (Fig 7B). This observation is consistent with a previous study in cattle showing a positive correlation between faecal *C*. *perfringens* levels and the acute-phase protein C-reactive protein (CRP) in blood plasma, although haptoglobin levels were not affected [31]. It should be noted that an increase in haptoglobin was observed only in animals with >0.4% *Clostridiaceae* 1 family (75^th^ percentile), and that within this fraction four out of eight animals had haptoglobin levels that were four fold higher than the average of the CON group. Interestingly, another recent study reported a decrease in haptoglobin expression levels following antibiotic treatment (mixture of ampicillin, gentamycin and metronidazole) of pre-term pigs, which was suggested to be due to decreased density of the gut microbiota [32].

Our study showed that both VAN and CTX caused a decrease in intestinal permeability of the 4 kDa FITC-dextran molecule, thus indicating a strengthening of integrity (Fig 5A). Permeability of this relatively large molecule is proposed to be via the ‘leak’ pathway in the tight junction protein complex [17]. Alterations in the ‘pore’ pathway, where smaller molecules and ions can pass, are therefore not detectable in the FITC-dextran assay. As expected, VAN, known to target Gram positive bacteria, reduced the relative abundance of several bacterial families within the Firmicutes and Actinobacteria phyla and increased the relative abundance of several Gram negative bacterial families within the Proteobacteria as well as *Verrucomicrobiaceae* consistent with previous studies [33]. However, a reduction of the Gram negative *Porphyromonadaceae* and notably also an increase in relative abundance of the Gram positive *Lactobacillaceae* were observed (Fig 3). The latter of these is consistent with the observed decrease in intestinal permeability as *Lactobacillu*s spp. have previously been shown to increase intestinal integrity in *in vitro* models [34–36]. The relative abundance of Proteobacteria was also generally increased by VAN, which could lead to increased levels of lipopolysaccharide (LPS) to cross the intestinal barrier and cause inflammation [13], however no increase in haptoglobin levels was seen in VAN group (Fig 5B). Finally, bacterial families within the Firmicutes were reduced by VAN including *Ruminococcaceae* and *Lachnospiraceae*, which belong to the butyrate producing *Clostridium* clusters IV and XIVa [37]. This reduction could explain the increase in pH in the caecum. Previously butyrate has been shown to decrease intestinal permeability in *in vitro* and animal disease models [38,39]. The reduction of butyrate producing bacteria did, however not increase the intestinal permeability in VAN treated animals in the present study.

For animals dosed with CTX we observed fewer significant changes in relative abundance of bacterial families than for VAN, however an increase in *Bifidobacterium* spp. was found as has previously been reported for rifaximin [40]. This is consistent with the observed positive effect on permeability, as *Bifidobacterium* spp. have also been shown to increase integrity in both *in vitro* and *in vivo* models [41–45] however in the current study CTX additionally increased the level of haptoglobin in the blood plasma indicating an increased level of inflammation (Fig 5B).

The antibiotic MTZ caused an increase in the intestinal permeability of FITC-dextran, but no change in the microbiota composition was detected. This indicates that MTZ may cause alterations to the intestinal permeability independent of the gut microbiota. Others have shown that MTZ can affect intestinal integrity in mice, indicated by a decrease in the mucus thickness, down regulation of *Muc2*, *TFF3*, and *Relmβ* gene expression and changes in the colonic microbiota [12].

The intestinal permeability assessed by the FITC-dextran assay was not affected by AMX, however increased levels of plasma haptoglobin were found in this group. This is interesting since AMX resulted in major changes in the bacterial composition and diversity, including reduction of butyrate producing bacteria (*Ruminococcaceae* and *Lachnospiraceae*) as well as increase of *Enterobacteriaceae* (Figs 2 and 3). Additionally, enlargement of the caecum was observed in the AMX and VAN groups (Fig 1D), which is consistent with previous observations in both germ-free mice [46] and antibiotic treated animals [47].

In conclusion, antibiotic induced changes in microbiota could be linked to intestinal permeability, although changes in permeability did not always result from major changes in microbiota and *vice versa*. The observed varying effects of different classes of antibiotics on intestinal integrity warrant further investigation and could potentially be important during selection of appropriate treatment for bacterial infections.

## Supporting Information

S1 Fig Principal component analysis (PCA) of the relative abundances of detected bacterial families in caecal and faecal samples. (A) The score plot shows samples grouped according to treatment groups CON (black), AMX (blue), CTX (green), VAN (yellow) and MTZ (red), with six animals in each group. ○ Caecal samples and + Faeces samples. (B) Loading plot indicating each of the bacterial families colored according to phylum. Firm, Firmicutes (blue); Bact, Bacteroidetes (red); Prot, Proteobacteria (green), Acti, Actinobacteria (yellow); Tene, Tenericutes (orange); Defe, deferribacteres (pink) and Verr, Verrucomicrobia (lilac).
(EPS)

S2 Fig Principal component analysis (PCA) of the relative abundances of detected bacterial families in ileum samples. (A) The score plot shows samples grouped according to treatment groups CON (black), AMX (blue), CTX (green), VAN (yellow) and MTZ (red), with six animals in each group. (B) Loading plot indicating each of the bacterial families colored according to phylum. Firm, Firmicutes (blue); Bact, Bacteroidetes (red); Prot, Proteobacteria (green), Acti, Actinobacteria (yellow); Tene, Tenericutes (orange); Defe, deferribacteres (pink); Verr, Verrucomicrobia (lilac) and Cyan, Cyanobacteria (cyan).
(EPS)

S3 Fig Gene expression of intestinal permeability markers. Mean relative gene expression of (A) claudin-1, (B) occludin, (C) ZO-1 and (D) *Muc2* in tissue samples obtained from the colon of animals in CON,control (grey); AMX, amoxicillin (blue); CTX, cefotaxime (green); VAN, vancomycin (yellow) and MTZ, Metronidazole (red) groups. Error bars show SEM. No significant differences between antibiotic groups and the control group were found.
(EPS)

## References

[1] Maynard CL, Elson CO, Hatton RD, Weaver CT. Reciprocal interactions of the intestinal microbiota and immune system. Nature. 2012;489: 231–41. 10.1038/nature11551 22972296 PMC4492337

[2] Cani PD, Possemiers S, Van de Wiele T, Guiot Y, Everard A, Rottier O, et al Changes in gut microbiota control inflammation in obese mice through a mechanism involving GLP-2-driven improvement of gut permeability. Gut. 2009;58: 1091–103. 10.1136/gut.2008.165886 19240062 PMC2702831

[3] Lewis K. Platforms for antibiotic discovery. Nat Rev Drug Discov. 2013;12: 371–87. 10.1038/nrd3975 23629505

[4] Jernberg C, Löfmark S, Edlund C, Jansson JK. Long-term impacts of antibiotic exposure on the human intestinal microbiota. Microbiology. 2010;156: 3216–23. 10.1099/mic.0.040618-0 20705661

[5] Ubeda C, Pamer EG. Antibiotics, microbiota, and immune defense. Trends Immunol. 2012;33: 459–66. 10.1016/j.it.2012.05.003 22677185 PMC3427468

[6] Pérez-Cobas AE, Artacho A, Knecht H, Ferrús ML, Friedrichs A, Ott SJ, et al Differential effects of antibiotic therapy on the structure and function of human gut microbiota. PLoS One. 2013;8: e80201 10.1371/journal.pone.0080201 24282523 PMC3839934

[7] Cho I, Yamanishi S, Cox L, Methé BA, Zavadil J, Li K, et al Antibiotics in early life alter the murine colonic microbiome and adiposity. Nature. 2012;488: 621–626. 10.1038/nature11400 22914093 PMC3553221

[8] Zhang Y, Limaye PB, Renaud HJ, Klaassen CD. Effect of various antibiotics on modulation of intestinal microbiota and bile acid profile in mice. Toxicol Appl Pharmacol. 2014;277: 138–45. 10.1016/j.taap.2014.03.009 24657338 PMC5533088

[9] Panda S, El khader I, Casellas F, López Vivancos J, García Cors M, Santiago A, et al Short-term effect of antibiotics on human gut microbiota. PLoS One. 2014;9: e95476 10.1371/journal.pone.0095476 24748167 PMC3991704

[10] Macfarlane S. Antibiotic treatments and microbes in the gut. Environ Microbiol. 2014;16: 919–24. 10.1111/1462-2920.12399 24471523

[11] Fujimura KE, Slusher NA, Cabana MD, Lynch SV. Role of the gut microbiota in defining human health. Expert Rev Anti Infect Ther. 2010;8: 435–54. 10.1586/eri.10.14 20377338 PMC2881665

[12] Wlodarska M, Willing B, Keeney KM, Menendez A, Bergstrom KS, Gill N, et al Antibiotic treatment alters the colonic mucus layer and predisposes the host to exacerbated *Citrobacter rodentium*-induced colitis. Infect Immun. 2011;79: 1536–45. 10.1128/IAI.01104-10 21321077 PMC3067531

[13] Cani PD, Bibiloni R, Knauf C, Neyrinck AM, Delzenne NM. Changes in gut microbiota control metabolic diet–induced obesity and diabetes in mice. Diabetes. 2008;57: 1470–81. 10.2337/db07-1403 18305141

[14] Fåk F, Ahrné S, Molin G, Jeppsson B, Weström B. Microbial manipulation of the rat dam changes bacterial colonization and alters properties of the gut in her offspring. Am J Physiol Gastrointest Liver Physiol. 2008;294: G148–54. 17962363 10.1152/ajpgi.00023.2007

[15] Hviid A, Svanström H, Frisch M. Antibiotic use and inflammatory bowel diseases in childhood. Gut. 2011;60: 49–54. 10.1136/gut.2010.219683 20966024

[16] John LJ, Fromm M, Schulzke J-DD. Epithelial Barriers in Intestinal Inflammation. Antioxid Redox Signal. 2011;15: 1255–1270. 10.1089/ars.2011.3892 21294654

[17] Shen L, Weber CR, Raleigh DR, Yu D, Turner JR. Tight junction pore and leak pathways: a dynamic duo. Annu Rev Physiol. 2011;73: 283–309. 10.1146/annurev-physiol-012110-142150 20936941 PMC4655434

[18] Antonopoulos DA, Huse SM, Morrison HG, Schmidt TM, Sogin ML, Young VB. Reproducible community dynamics of the gastrointestinal microbiota following antibiotic perturbation. Infect Immun. 2009;77: 2367–2375. 10.1128/IAI.01520-08 19307217 PMC2687343

[19] Christensen E, Licht T, Leser T, Bahl M. Dietary Xylo-oligosaccharide stimulates intestinal bifidobacteria and lactobacilli but has limited effect on intestinal integrity in rats. BMC Res Notes. 2014;7: 660 10.1186/1756-0500-7-660 25238818 PMC4179812

[20] Wang Q, Garrity GM, Tiedje JM, Cole JR. Naive Bayesian classifier for rapid assignment of rRNA sequences into the new bacterial taxonomy. Appl Environ Microbiol. 2007;73: 5261–7. 17586664 10.1128/AEM.00062-07 PMC1950982

[21] Claesson MJ, O’Sullivan O, Wang Q, Nikkilä J, Marchesi JR, Smidt H, et al Comparative analysis of pyrosequencing and a phylogenetic microarray for exploring microbial community structures in the human distal intestine. PLoS One. 2009;4: e6669 10.1371/journal.pone.0006669 19693277 PMC2725325

[22] Nejrup RG, Bahl MI, Vigsnæs LK, Heerup C, Licht TR, Hellgren LI. Lipid hydrolysis products affect the composition of infant gut microbial communities in vitro. Br J Nutr. 2015; 1–12. 10.1017/S0007114515000811 25992463

[23] Canibe N, Højberg O, Badsberg JH, Jensen BB. Effect of feeding fermented liquid feed and fermented grain on gastrointestinal ecology and growth performance in piglets. J Anim Sci. 2007;85: 2959–2971. 17591711 10.2527/jas.2006-744

[24] Jensen MT, Cox RP, Jensen BB. Microbial production of skatole in the hind gut of pigs given different diets and its relation to skatole deposition in backfat. Anim Sci. 1995;61: 293–304.

[25] White JR, Nagarajan N, Pop M. Statistical methods for detecting differentially abundant features in clinical metagenomic samples. PLoS Comput Biol. 2009;5: e1000352 10.1371/journal.pcbi.1000352 19360128 PMC2661018

[26] Edgar RC. Search and clustering orders of magnitude faster than BLAST. Bioinformatics. 2010;26: 2460–1. 10.1093/bioinformatics/btq461 20709691

[27] Caporaso JG, Kuczynski J, Stombaugh J, Bittinger K, Bushman FD, Costello EK, et al QIIME allows analysis of high-throughput community sequencing data. Nat Methods. 2010;7: 335–6. 10.1038/nmeth.f.303 20383131 PMC3156573

[28] Löfmark S, Edlund C, Nord CE. Metronidazole is still the drug of choice for treatment of anaerobic infections. Clin Infect Dis. 2010;50 Suppl 1: S16–23. 10.1086/647939 20067388

[29] Louis P, Hold GL, Flint HJ. The gut microbiota, bacterial metabolites and colorectal cancer. Nat Rev Microbiol. 2014;12: 661–672. 10.1038/nrmicro3344 25198138

[30] Ferreyra JA, Wu KJ, Hryckowian AJ, Bouley DM, Weimer BC, Sonnenburg JL. Gut Microbiota-Produced Succinate Promotes *C*. *difficile* Infection after Antibiotic Treatment or Motility Disturbance. Cell Host Microbe. 2014;16: 770–777. 10.1016/j.chom.2014.11.003 25498344 PMC4859344

[31] Schroedl W, Kleessen B, Jaekel L, Shehata AA, Krueger M. Influence of the gut microbiota on blood acute-phase proteins. Scand J Immunol. 2014;79: 299–304. 10.1111/sji.12161 24498969

[32] Jiang P, Jensen ML, Cilieborg MS, Thymann T, Wan JM-F, Sit W-H, et al Antibiotics increase gut metabolism and antioxidant proteins and decrease acute phase response and necrotizing enterocolitis in preterm neonates. PLoS One. 2012;7: e44929 10.1371/journal.pone.0044929 23028687 PMC3441690

[33] Dubourg G, Lagier J-C, Armougom F, Robert C, Audoly G, Papazian L, et al High-level colonisation of the human gut by *Verrucomicrobia* following broad-spectrum antibiotic treatment. Int J Antimicrob Agents. 2013;41: 149–55. 10.1016/j.ijantimicag.2012.10.012 23294932

[34] Klingberg TD, Pedersen MH, Cencic A, Budde BB. Application of measurements of transepithelial electrical resistance of intestinal epithelial cell monolayers to evaluate probiotic activity. Appl Environ Microbiol. 2005;71: 7528–7530. 16269795 10.1128/AEM.71.11.7528-7530.2005 PMC1287686

[35] Anderson RC, Cookson AL, McNabb WC, Park Z, McCann MJ, Kelly WJ, et al *Lactobacillus plantarum* MB452 enhances the function of the intestinal barrier by increasing the expression levels of genes involved in tight junction formation. BMC Microbiol. 2010;10: 316 10.1186/1471-2180-10-316 21143932 PMC3004893

[36] Commane DM, Shortt CT, Silvi S, Cresci A, Hughes RM, Rowland IR. Effects of fermentation products of pro- and prebiotics on trans-epithelial electrical resistance in an *in vitro* model of the colon. Nutr Cancer. 2005;51: 102–109. 15749636 10.1207/s15327914nc5101_14

[37] Pryde SE, Duncan SH, Hold GL, Stewart CS, Flint HJ. The microbiology of butyrate formation in the human colon. FEMS Microbiol Lett. 2002;217: 133–9. 12480096 10.1111/j.1574-6968.2002.tb11467.x

[38] Wang H-BB, Wang P-YY, Wang X, Wan Y-LL, Liu Y-CC. Butyrate enhances intestinal epithelial barrier function via up-regulation of tight junction protein Claudin-1 transcription. Dig Dis Sci. 2012;57: 3126–35. 10.1007/s10620-012-2259-4 22684624

[39] Kanauchi O, Iwanaga T, Mitsuyama K, Saiki T, Tsuruta O, Noguchi K, et al Butyrate from bacterial fermentation of germinated barley foodstuff preserves intestinal barrier function in experimental colitis in the rat model. J Gastroenterol Hepatol. 1999;14: 880–8. 10535469 10.1046/j.1440-1746.1999.01971.x

[40] Maccaferri S, Vitali B, Klinder A, Kolida S, Ndagijimana M, Laghi L, et al Rifaximin modulates the colonic microbiota of patients with Crohn’s disease: an *in vitro* approach using a continuous culture colonic model system. J Antimicrob Chemother. 2010;65: 2556–65. 10.1093/jac/dkq345 20852272

[41] Ewaschuk JB, Diaz H, Meddings L, Diederichs B, Dmytrash A, Backer J, et al Secreted bioactive factors from *Bifidobacterium infantis* enhance epithelial cell barrier function. Am J Physiol Gastrointest Liver Physiol. 2008;295: G1025–G1034. 10.1152/ajpgi.90227.2008 18787064

[42] Putaala H, Salusjärvi T, Nordström M, Saarinen M, Ouwehand AC, Bech Hansen E, et al Effect of four probiotic strains and *Escherichia coli* O157:H7 on tight junction integrity and cyclo-oxygenase expression. Res Microbiol. 2008;159: 692–8. 10.1016/j.resmic.2008.08.002 18783733

[43] López P, Monteserín DC, Gueimonde M, de los Reyes-Gavilán CG, Margolles A, Suárez A, et al Exopolysaccharide-producing *Bifidobacterium* strains elicit different *in vitro* responses upon interaction with human cells. Food Res Int. 2012;46: 99–107.

[44] Madsen K, Cornish A, Soper P, McKaigney C, Jijon H, Yachimec C, et al Probiotic bacteria enhance murine and human intestinal epithelial barrier function. Gastroenterology. 2001;121: 580–591. 11522742 10.1053/gast.2001.27224

[45] Bergmann KR, Liu SXL, Tian R, Kushnir A, Turner JR, Li H-LL, et al Bifidobacteria stabilize claudins at tight junctions and prevent intestinal barrier dysfunction in mouse necrotizing enterocolitis. Am J Pathol. 2013;182: 1595–1606. 10.1016/j.ajpath.2013.01.013 23470164 PMC3644725

[46] Hill DA, Hoffmann C, Abt MC, Du Y, Kobuley D, Kirn TJ, et al Metagenomic analyses reveal antibiotic-induced temporal and spatial changes in intestinal microbiota with associated alterations in immune cell homeostasis. Mucosal Immunol. 2010;3: 148–58. 10.1038/mi.2009.132 19940845 PMC2824244

[47] Reikvam DH, Erofeev A, Sandvik A, Grcic V, Jahnsen FL, Gaustad P, et al Depletion of murine intestinal microbiota: effects on gut mucosa and epithelial gene expression. PLoS One. 2011;6: e17996 10.1371/journal.pone.0017996 21445311 PMC3061881

